# Machine learning-based causal models for predicting the response of individual patients to dexamethasone treatment as prophylactic antiemetic

**DOI:** 10.1038/s41598-023-34505-0

**Published:** 2023-05-09

**Authors:** Taisuke Mizuguchi, Shigehito Sawamura

**Affiliations:** Department of Anesthesia, Teikyo University, 2-11-1 Kaga, Itabashi-ku, Tokyo, 173-8606 Japan

**Keywords:** Medical research, Epidemiology, Nausea, Vomiting, Biomedical engineering, Computational science, Machine learning, Predictive medicine, Statistical methods

## Abstract

Risk-based strategies are widely used for decision making in the prophylaxis of postoperative nausea and vomiting (PONV), a major complication of general anesthesia. However, whether risk is associated with individual treatment effect remains uncertain. Here, we used machine learning-based algorithms for estimating the conditional average treatment effect (CATE) (double machine learning [DML], doubly robust [DR] learner, forest DML, and generalized random forest) to predict the treatment response heterogeneity of dexamethasone, the first choice for prophylactic antiemetics. Electronic health record data of 2026 adult patients who underwent general anesthesia from January to June 2020 were analyzed. The results indicated that only a small subset of patients respond to dexamethasone treatment, and many patients may be non-responders. Estimated CATE did not correlate with predicted risk, suggesting that risk may not be associated with individual treatment responses. The current study suggests that predicting treatment responders by CATE models may be more appropriate for clinical decision making than conventional risk-based strategy.

## Introduction

Postoperative nausea and vomiting (PONV) are major complications of general anesthesia, leading to significant patient discomfort^1^, increased health care costs^2^, and unanticipated side effects^3,4^. Although interventions such as antiemetic treatment and avoidance of risk factors are suggested for PONV prophylaxis^5,6^, the evidence is derived from the average treatment effect of the population. In practice, treatment effects can be heterogeneous, and interventions may be ineffective or even harmful in a subset of patients. Identifying individuals who respond to treatment is essential to avoid unnecessary interventions that increase the risk of adverse events.

The individual treatment effect measures the difference in outcomes for the same individual in alternative futures, with or without treatment^7^. Because such observation is impossible, predicted risk has been a popular surrogate index for treatment decision making in clinical practice. Many clinical guidelines, including those for PONV management, recommend risk-tailored prophylaxis^5,8^ which is determined by a risk score or a prediction model^9,10^. Most of the existing research on treatment decision has focused on developing accurate prediction models^11,12^ and optimizing risk-tailored prophylaxis strategy^13–15^. However, conventional risk-based strategies still cannot avoid unnecessary intervention, because the predicted risk may not be associated with the heterogeneity of treatment response.

Recent advancements in methodologies have provided tools to directly estimate individual treatment effects^16–21^, but there are still limited reports on their clinical application^22–26^. In this study, we predicted the treatment effect heterogeneity of dexamethasone, the first choice of prophylactic antiemetics^5^, by applying machine learning-based causal models for observational data^16–20^. These models estimate the conditional average treatment effect (CATE) in a subpopulation of patients characterized by a combination of covariates. Furthermore, we evaluated the factors associated with treatment response heterogeneity by applying Shapley additive explanations (SHAP) method^27–29^ to CATE models. The main contribution of this research is the proposal of a framework for the prediction of the treatment response heterogeneity of dexamethasone for PONV prophylaxis, which may improve the quality of PONV management.

## Results

### Population

Of the 2026 patient data used for analysis, 756 (37.3%) were treated with dexamethasone. In the training/validation set (n = 1219; 60.2%; January–March 2020), the median (standard deviation [SD]) age was 58.5 (19.1) years, 581 (47.7%) were female, 438 (35.9%) were treated with dexamethasone, and 290 (23.8%) experienced PONV. In the test set (n = 807; 39.8%; April–June 2020), the median (SD) age was 59.7 (18.2) years, 398 (49.3%) were female, 318 (39.4%) were treated with dexamethasone, and 290 (23.8%) experienced PONV. The baseline characteristics of the two datasets were broadly similar, except for the decreased number of surgeries (training/validation set, 1219; test set, 807; 33.8% decrease) and intensive care unit (ICU) admissions (training/validation set, 310; test set, 141; 54.5% decrease) in the test set due to coronavirus pandemic (Table 1). The category distribution of PONV is presented in Supplementary Table 1.

**Table 1:** Baseline patient characteristics of overall dataset, training/validation set, and test set^a^.

	Overall (n = 2026)	Training/validation (n = 1219)	Test (n = 807)
PONV	477 (23.5)	290 (23.8)	187 (23.2)
Age, mean (SD), years	58.5 (19.1)	57.7 (19.6)	59.7 (18.2)
Sex (female)	979 (48.3)	581 (47.7)	398 (49.3)
Non smoker	1090 (53.8)	674 (55.3)	416 (51.5)
PONV or motion sickness history	194 (9.6)	114 (9.4)	80 (9.9)
Hypertension	661 (32.6)	375 (30.8)	286 (35.4)
Diabetes	306 (15.1)	184 (15.1)	122 (15.1)
Psychiatric disease	102 (5.0)	69 (5.7)	33 (4.1)
Malignancy	596 (29.4)	321 (26.3)	275 (34.1)
History of stroke	162 (8.0)	107 (8.8)	55 (6.8)
Asthema	91 (4.5)	55 (4.5)	36 (4.5)
COPD	194 (9.6)	149 (12.2)	45 (5.6)
Coronary disease	49 (2.4)	26 (2.1)	23 (2.9)
Post PCI or CABG	83 (4.1)	51 (4.2)	32 (4.0)
Asynergy	63 (3.1)	36 (3.0)	27 (3.3)
ASA-PS 1	557 (27.5)	371 (30.4)	186 (23.0)
ASA-PS 2	1233 (60.9)	689 (56.5)	544 (67.4)
ASA-PS 3	236 (11.6)	159 (13.0)	77 (9.5)
Anesthesia time, mean (SD), hours	3.4 (2.0)	3.3 (2.0)	3.5 (2.0)
TIVA	266 (13.1)	143 (11.7)	123 (15.2)
Peripheral nerve block	328 (16.2)	177 (14.5)	151 (18.7)
Epidural anesthesia	265 (13.1)	145 (11.9)	120 (14.9)
Continuous opioid infusion	704 (34.7)	415 (34.0)	289 (35.8)
Droperidol bolus	238 (11.7)	131 (10.7)	107 (13.3)
Dexamethasone bolus	756 (37.3)	438 (35.9)	318 (39.4)
Elective surgery	1809 (89.3)	1099 (90.2)	710 (88.0)
Emergency surgery	217 (10.7)	120 (9.8)	97 (12.0)
ICU admission	451 (22.3)	310 (25.4)	141 (17.5)

*PONV* postoperative nausea and vomiting, *COPD* chronic obstructive pulmonary disease, *PCI* percutaneous coronary intervention, *CABG* coronary artery bypass graft, *ASA-PS* American Society of Anesthesiologists Physical Status, *TIVA* total intravenous anesthesia, *ICU* intensive care unit.

^a^Data are expressed as No. (%) unless otherwise indicated.

### Conditional average treatment effect models

**Figure 1:**
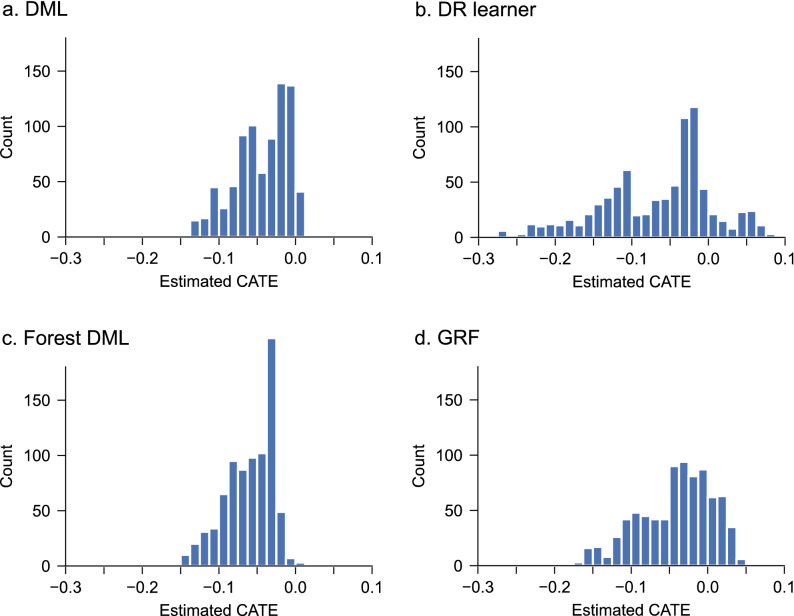
Distribution of the estimated CATE. *CATE* conditional average treatment effect, *DML* double machine learning, *DR* doubly robust, *GRF* generalized random forest.

**Figure 2:**
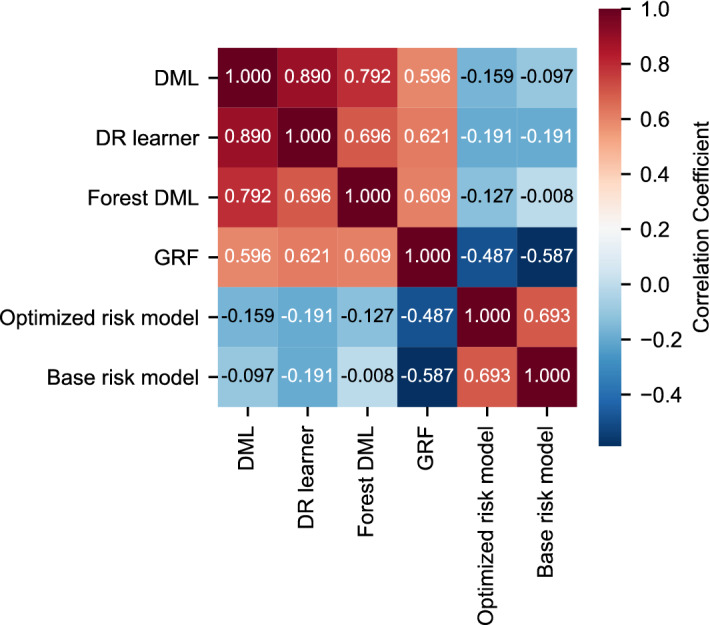
Correlation coefficient maps for the Estimated treatment effect and the predicted risk. Color-coding and the number in each cell represent the Pearson correlation coefficient between the model estimates. *DML* double machine learning, *DR* doubly robust, *GRF* generalized random forest.

We predicted the individualized treatment response to dexamethasone for PONV prophylaxis using multiple CATE estimation algorithms (double machine learning [DML]^16^, doubly robust [DR] learner^17,18^, generalized random forests [GRF]^19^, and forest DML^20^). The distributions of the estimated CATE, which reflects the treatment response, were skewed to negative values (Fig. 1), consistent with PONV risk reduction by dexamethasone^6^. In all models, CATE distribution had a peak near zero, corresponding to the non-responders. The estimated CATE values showed a strong correlation among the different models, suggesting the reproducibility of the estimates in different algorithms (Fig. 2).

For comparison, we also created two machine learning-based risk prediction models: (1) a model with covariates selected by stepwise selection (optimized risk model) and (2) a model with previously reported risk factors as covariates (base risk model). In terms of risk prediction, the performance of the optimized risk model (area under the receiver operating characteristic curve [AUROC] 0.714; 95% CI 0.683–0.746) was among the best of previously reported models^9^ and outperformed the base risk model using previously reported risk factors as covariates (AUROC 0.635; 95% CI 0.602–0.668). The performance indicators of the risk prediction models are provided in Supplementary Table 2. However, there was a small or no correlation between the predicted risk and the estimated CATE (Fig. 2), suggesting a lack of association between risk and treatment response.

### Clinical implication

**Figure 3:**
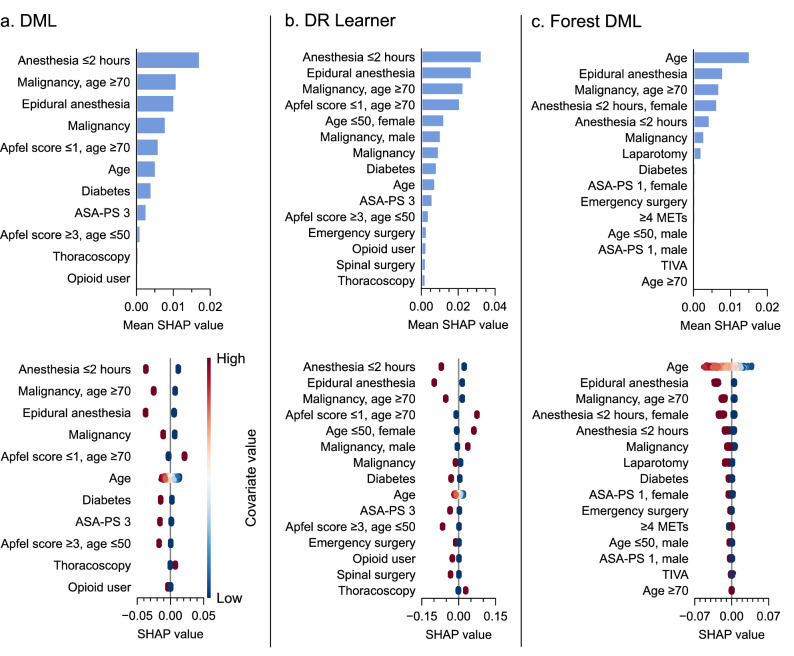
Covariate importance determined by SHAP values of CATE estimation models. The bar chart in the top row displays the global importance of the covariates on conditional average treatment effect (CATE) estimation, represented as the mean absolute Shapley additive explanations (SHAP) value of the covariates over all the given samples. The strip plot in the bottom row displays the change in the estimated CATE value associated with the covariate. Each dots represent an individual, piled up along the row to show density. Binary covariates are displayed as either 1 (High) or 0 (Low). *DML* double machine learning, *DR* doubly robust, *ASA-PS* American Society of Anesthesiologists Physical Status, *METs* metabolic equivalents, *TIVA* total intravenous anesthesia.

The importance of the covariates in CATE estimation was assessed using the Shapley additive explanations (SHAP)^27^ value (Fig. 3). Among the key contributors, anesthesia duration of ≤ 2 h, epidural anesthesia, malignancy, and old age changed the model estimate toward negative values. Conversely, young age and Apfel score of ≤ 1 in those aged over 70 years changed the model estimate toward positive values.

### Goodness-of-fit analysis

The propensity score used in the CATE model was predicted by L2-regularized logistic regression (AUROC 0.751; 95% CI 0.722–0.781). This score was mainly within the range of 0.05–0.95, suggesting reasonable overlap across the treatment and untreated groups (Supplementary Fig. 1).

### Uplift curve evaluation

The model performance in identifying responders to treatment was evaluated using the area under the uplift curve (AUUC)^30,31^. A greater positive AUUC indicates better model performance, and the AUUC of a null model is zero. The AUUC of the CATE models was significantly greater than zero in DML (AUUC 22.4; 95% confidence interval [CI] 6.4–38.5) and DR learner (AUUC 18.4; 95% CI 2.0–34.5) (Supplementary Fig. 2), indicating that the models could identify the responders to prophylactic dexamethasone treatment. Sensitivity analysis also supported the results from the uplift curve evaluation of the CATE model (Supplementary Figs. 3, 4, and 5).

## Discussion

This retrospective cohort study identified the individuals likely to respond to dexamethasone treatment for PONV prophylaxis using CATE models, and only a small subset of patients may respond to the treatment. Furthermore, predicted risks were not associated with the estimated treatment responses.

Although randomized control trials (RCTs) are considered the gold standard of evidence-based medicine, they cannot determine whether a treatment is beneficial to a specific individual. In practice, many clinical guidelines recommend risk-based patient selection for intervention^5,8^. Risk-stratified subgroup analyses in other clinical disciplines have suggested an association between risk and treatment effect^32–34^. However, a careful interpretation is required for subgroup analyses, because insufficient power in stratified samples can result in misleading results^35^.

In the current study, the results indicated that predicted risk may lack association with individual treatment responses. Prior studies in nephrology^22^ and critical care^24^ report that treatment effect heterogeneity models are superior to the risk prediction models in identifying the responder to the treatment, supporting the current results. Furthermore, our results indicate that many patients may be non-responders to dexamethasone treatment. Thus, direct prediction of treatment heterogeneity may be more efficient than conventional risk-based strategy.

Previously reported clinical applications of CATE models estimated the treatment effect from the difference in the predicted risk between the treatment groups in RCT data^22,24,36^. The methods applied in the current study were designed for observational data, with confounder adjustments intrinsic to the algorithm^16–20^. Modeling treatment effect heterogeneity in observational data has a significant advantage, enabling the use of large databases, such as electronic health records, that capture a broad and diverse population.

This study has several limitations. First, the accuracy of the CATE model cannot be evaluated directly because the ground truth is unobservable. Thus, we have evaluated the CATE models using surrogate measures of evaluation, including the reproducibility of the results among different CATE algorithms. However, we still cannot rule out the possibility of using biased measures for evaluation, which are discussed in further detail below. Second, we used observational data, and the estimates of individual treatment responses obtained by the CATE models can be biased if the identifying assumptions were violated. CATE models use the propensity score model to adjust for the confounders, and we have selected observable potential confounders for the covariates, including previously reported risk factors of PONV. The distribution range of the propensity score suggested that the positivity assumption holds. Furthermore, the timing of dexamethasone administration was always at anesthesia induction, and a previous meta-analysis reported that there was no significant difference in the treatment effect within the dosage used in this study (4–8 mg)^37^. Thus we assumed that Stable Unit Treatment Value Assumption (SUTVA) holds. Although we cannot completely rule out the influence of unobserved confounders, we consider that the identifying assumptions were reasonably satisfied. Third, results may not be generalizable to all patients because our data were derived from a single institution. We performed validation of our model by temporal splitting of the data^38^. Furthermore, our data include the coronavirus pandemic period, and the splitting point is in proximity to the first declaration of a state of emergency in Tokyo. Our results should be more generalizable than conventional temporal splitting, considering the environmental changes caused by coronavirus in the test set. Fourth, there may be selection bias because we excluded some samples, such as intubated patients, in which we could not evaluate the outcome.

The current results demonstrate a framework for identifying the responders to antiemetic dexamethasone treatment by applying machine learning-based causal models. Conventional risk prediction models may not be suitable for identifying a small subset of treatment responders, and the approach using CATE models may be a powerful tool for optimizing PONV management. Further prospective validation is needed for application in clinical practice.

## Methods

### Ethics

All data were extracted from institutional electronic health records after approval by the Ethics Committee of Teikyo University Hospital. All methods were carried out in accordance with the institutional guidelines and regulations. Informed consent was obtained from all the participants in the form of opt-out on the website. De-identified data were used for analysis.

### Datasets

This study followed the Strengthening the Reporting of Observational Studies in Epidemiology (STROBE) reporting guideline^39^. Analyses were based on a retrospective cohort of adult patients (age ≥ 18 years) who underwent general anesthesia at Teikyo University Hospital from January 2020 to June 2020. Extracted electronic health record data included demographics, routine preoperative evaluation, anesthesia records, and routine postoperative evaluation (Supplementary Table 3). Supplementary Fig. 6 shows the study flowchart. Exclusion criteria were open-heart cardiac surgery and intubated or unconscious patients. Patients without extubation or unexpected events within 24 h of surgery (emergency re-operation, intubation, intensive care unit [ICU] admission, or patient escape) precluding outcome observation were regarded as censored data and were excluded. Patients discharged on postoperative day 1, within 24 h of surgery, were included in the analysis. No patient was discharged on postoperative day 0. Three patients with missing data (anesthesia duration, 0.15%) and 18 patients with dexamethasone treatment dose < 4 mg (0.89%) were also excluded from the analysis.

Data were split temporally into the training/validation set (January–March 2020) and the test set (April–June 2020). A state of emergency was declared in Tokyo on April 7, 2020, due to the coronavirus pandemic. Less urgent elective surgery and postsurgical ICU admission were restricted, and the environment changed dramatically from infection control procedures in the test set. We chose temporal splitting to show the generalizability of the CATE models in temporally different datasets^38^. Random, non-temporal splitting of the datasets was used for sensitivity analysis. For cross-validation, the training/validation set was further randomly split into the training set and the validation set.

### Primary outcome

Outcome *Y* of interest was nausea or vomiting within 24 h of surgery, assessed on a binary scale. The assessment was based on routine postoperative evaluation by anesthesiologists on postoperative day 1 and routine nurse evaluation in the postanesthesia care unit, ICU, or general ward.

### Treatment

The treatment *T* considered was an intravenous bolus of 4–8 mg dexamethasone at anesthesia induction on a binary scale, which is the recommended dose in the guideline of PONV management^5^. A previous meta-analysis indicated no significant difference in the incidence of PONV between 4–5 mg and 8–10 mg dexamethasone treatment^37^.

### Conditional average treatment effect models

CATE models use machine learning algorithms to estimate treatment effect, conditional on the combination of covariates reflecting patient characteristics^40^. There is no currently accepted standard algorithm for CATE estimation. Thus, we evaluated multiple algorithms (double machine learning [DML]^16^, doubly robust [DR] learner^17,18^, generalized random forests [GRF]^19^, and forest DML^20^) and compared their performance with the risk prediction models. Let *Y* denote the outcome of interest, *T* denote the treatment,* X* denote the covariates characterizing the individuals, and *W* denote the observed confounders.

We used L2-regularized logistic regression for the nuisance parameter estimation, except for GRF, which is designed to use random forest. L2 regularization adds a penalty term weighted by the square of the coefficient to avoid overfitting. Ridge regression, which is an L2-regularized linear regression, was used for the final stage regression model in DML and DR learner.

#### Double machine learning (DML)

This algorithm estimates CATE *θ*(*X*) by combining the outcome prediction model and propensity score model into a residual-on-residual regression. Machine learning models are susceptible to two sources of estimation bias: regularization and overfitting. DML implements a solution for this problem by correcting regularization bias by Neyman orthogonality and overfitting via sample splitting. The following partially linear model is assumed:

$$Y = \theta (X)T + g(X,\;W) + \varepsilon ,\quad E[\varepsilon |X,\;W] = 0,$$

$$T = e(X,\;W) + \kappa ,\quad E[\kappa |X,\;W] = 0.$$

*g*(*X*, *W*) is an arbitrary function for estimating the outcome variable *Y*, *e*(*X*, *W*) is a propensity score model, and *m*(*X*, *W*) is a risk prediction model. ε and κ are error coefficients. The samples are split into *K* subsamples, then *m*(*X*, *W*) = E[*Y* | *X*, *W*] and *e*(*X*, *W*) = E[*T* | *X*, *W*] are predicted in each subsample by arbitrary machine learning models. These nuisance parameters are used to create a residuals-on-residuals regression model:

$$Y - m(X,\;W) = \theta (X)(T - e(X,\;W)) + \varepsilon .$$

The score function $\psi$ is defined as a dot product of the error term of the residuals-on-residuals regression and the error term of the propensity score model *e*(*X*, *W*):

$$\psi (Z;\;\theta ,\;h(X,\;W)) = (Y - m(X,\;W) - \theta (X)(T - e(X,\;W))) \cdot (T - e(X,\;W)),$$

where the observed parameters *Z* = {*Y*, *T*, *X*, *W*} and nuisance parameters *h* = {*m* (*X*, *W*), *e*(X, *W*)}. The moment condition^41^ is satisfied when the score function is zero, indicating that the two error terms are uncorrelated. The estimator $\tilde{\theta }$ (*X*) is constructed as the solution to

$$\frac{1}{K}\sum\limits_{k = 1}^{K} {\sum\limits_{i = 1}^{n} {\psi \left( {Z_{i} ;\;\theta ,\;\hat{h}(X_{i} ,\;W_{i} )} \right) = 0} } .$$

Estimated CATE $\tilde{\theta }$ minimizes the average of expected score functions across all *K* subsamples.

#### Doubly robust (DR) learner

This algorithm is a modified version of conventional doubly robust approach^42^, and estimates CATE *θ*(*X*) using the outcome prediction model and propensity score model as the nuisance parameters. Parameters conditional to each treatment level are defined for binary treatment *T* = *t* ∈ {0, 1}: potential outcome *Y*^*t*^, risk prediction model *m*_*t*_(*X*, *W*), propensity score model *e*_*t*_(*X*, *W*), and error coefficient *γ*_*t*_. The following models are assumed:

$$Y^{t} = m_{t} (X,\;W) + \gamma_{t} ,\quad E[\gamma |X,\;W] = 0,$$

$$\Pr [T = t|X,\;W] = e_{t} (X,\;W).$$

Independent and identically distributed samples labeled *i* = 0, 1, …, *n*, each consisting of the following parameters are defined: the outcome *Y*_*i*_ ∈ ${\mathbb{R}}$, the treatment *T*_*i*_ ∈ {0, 1}, the covariates *X*_*i*_ ∈ ${\mathbb{R}}$, and the observed confounders *W*_*i*_ ∈ ${\mathbb{R}}$. The following estimates of potential outcomes $Y_{i,t}^{DR}$ are constructed for *t* = 0 and *t* = 1:

$$Y_{i,t}^{DR} = m_{t} (X_{i} ,\;W_{i} ) + \frac{{Y_{i} - m_{t} (X_{i} ,\;W_{i} )}}{{e_{t} (X_{i} ,\;W_{i} )}} \cdot 1\{ T_{i} = t\} .$$

CATE *θ*(*X*) is estimated by solving the regression model over a parameter target class $\Theta$:

$$\arg \mathop {\min }\limits_{\theta \in \Theta } E\left[ {\left( {\hat{Y}_{i,1}^{DR} - \hat{Y}_{i,0}^{DR} - \theta (X)} \right)^{2} } \right].$$

#### Generalized random forest (GRF)

This algorithm estimates CATE from the local regression of moment equation^41^, using data-adapted weight obtained from the modified random forest with splitting criteria which maximizes heterogeneity. The confounders are adjusted by residualizing the outcome *Y* and the treatment *T*, using predicted outcome *m*(*X*, *W*) = E[*Y* | *X*, *W*] and propensity score *e*(*X*, *W*) = E[*T* | *X*, *W*] as the nuisance parameters. These parameters are predicted by conventional random forest. Subsequent steps are performed on residualized data instead of the original. Subsamples are chosen randomly from the sample without replacement, then split into equal size samples for the splitting phase and estimation phase. Such partitioning is called "honest" when the information used for splitting is never used for estimation. Honesty avoids overfitting and ensures statistical inference. In the splitting phase, the causal tree is grown by splitting the sample space, maximizing the heterogeneity of the estimated treatment effect between the child nodes. Numerical approximations of heterogeneity based on gradient tree algorithms are made to reduce computational costs. The terminal node in which each sample fall represents a cluster with similar propensity. In the estimation phase, samples are fitted to the causal tree to determine which terminal node it falls in. Data-adaptive weight *α* is calculated as a list of neighboring training samples, weighted by the frequency it fell in the same terminal node as the test sample. CATE $\tilde{\theta }$ is estimated by solving the weighted moment equation using this list of data-adaptive weight and the score function $\psi$:

$$\sum\limits_{i = 1}^{n} {\alpha_{i} (X)\psi \left( {Z_{i} ;\;\theta ,\;\hat{h}(X_{i} ,\;W_{i} )} \right) = 0} .$$

#### Forest double machine learning (DML)

This algorithm estimates CATE using the moment equation of DML in the splitting phase of GRF. The original study^20^ used local fitting of the nuisance parameters, but we used a modified version^40^ with the global fitting of nuisance parameters to reduce computational costs.

### Risk prediction models

We created two machine learning-based PONV risk prediction models to compare with CATE models: base risk model with previously reported risk factors of PONV^5,9^ (age, anesthesia duration, sex, history of motion sickness or PONV, nonsmoker, postsurgical opioid infusion) selected as covariates and optimized risk model with covariates chosen by stepwise selection. The covariates are provided in Supplementary Table 3. L2-regularized logistic regression was used for both models. We performed fivefold cross-validation, and the models with the highest area under the receiver operating characteristic curve (AUROC) were selected.

### Model interpretation

We used Shapley additive explanations (SHAP) to interpret the model estimation^27^. SHAP is a game theory-based approach to explain the output of a machine learning model. We used SHAP to assess the contribution of each covariate in the CATE estimation.

### Uplift curve evaluation

The accuracy of the CATE model cannot be evaluated directly because its true value is unobservable. Thus, we evaluated the models using the uplift curve, which is a popular metric for evaluating CATE models^30,43^. The samples were sorted by the rank of the estimated CATE values, and subsamples consisting of top *k* samples (*k* = 1, 2, …, *n*; *n*, total sample size) were created for each value of *k*. The uplift curve *f*(*k*) plots the estimated difference in PONV events between the treated and untreated groups, calculated from the observed outcome in each subsample:

$$f(k) = \left( {\frac{{\sum\nolimits_{i = 1}^{k} {T_{i} Y_{i} } }}{{\sum\nolimits_{i = 1}^{k} {T_{i} } }} - \frac{{\sum\nolimits_{i = 1}^{k} {(1 - T_{i} )Y_{i} } }}{{\sum\nolimits_{i = 1}^{k} {(1 - T_{i} )} }}} \right)k.$$

The baseline plots the expected uplift curve value when the subsamples consist of a random CATE:

$$baseline = \frac{f(n)}{n}k.$$

If the value of CATE is estimated correctly, a greater decrease in PONV events should be observed in the uplift curve compared to the baseline. For statistical analysis, the area under the uplift curve (AUUC)^30,31^ was calculated as the cumulative difference between the baseline and the uplift curve values:

$$AUUC = \frac{1}{2}f(n) - \frac{1}{n}\sum\limits_{k = 1}^{n} {f(k)} .$$

A greater positive AUUC indicates better model performance in identifying the responder to the treatment. The AUUC of a null model is zero. The 95% CI of the AUUC was estimated from 2000 bootstrap resampling.

The uplift curve was originally designed for the evaluation of randomized data^30,43,44^. We modified the approach by separately adjusting for confounders in all subsamples constituting the uplift curve. The confounders were adjusted using inverse probability weighting (IPW):

$$f(k) = \left( {\frac{{\sum\nolimits_{i = 1}^{k} {\frac{{T_{i} Y_{i} }}{{e(W_{i} )}}} }}{{\sum\nolimits_{i = 1}^{k} {\frac{{T_{i} }}{{e(W_{i} )}}} }} - \frac{{\sum\nolimits_{i = 1}^{k} {\frac{{(1 - T_{i} )Y_{i} }}{{(1 - e(W_{i} ))}}} }}{{\sum\nolimits_{i = 1}^{k} {\frac{{(1 - T_{i} )}}{{(1 - e(W_{i} ))}}} }}} \right)k$$

or a doubly robust estimator^42^:

$$f(k) = \sum\limits_{i = 1}^{k} {\left( {m_{1} (W_{i} ) + \frac{{T_{i} (Y_{i} - m_{1} (W_{i} ))}}{{e(W_{i} )}} - \left( {m_{0} (W_{i} ) + \frac{{(1 - T_{i} )(Y_{i} - m_{0} (W_{i} ))}}{{(1 - e(W_{i} ))}}} \right)} \right)} .$$

The propensity score *e*(*W*) and risk model conditional on treated *m*_1_(*W*) and untreated *m*_0_(*W*) were predicted by L2-regularized logistic regression, using observed confounders *W* as covariates^45^.

### Model parameter selection

The samples were split into the training/validation set and the test set. All procedures for model parameter selection were performed in the training/validation set, which was further split into the training set and the validation set for evaluation. Let *X* denote the covariates characterizing the individuals and *W* denote the observed confounders. The observed confounders *W* were selected from previously reported risk factors^5,9^ and expert opinions as fixed parameters. The parameters of CATE models, including covariates *X*, were selected by stepwise selection with threefold cross-validation, and those with the highest AUUC in the validation set were used. The parameters of risk prediction models were selected similarly, except fivefold cross-validation was used and AUROC was used for performance evaluation. Different fold cross-validation was used for CATE model and risk prediction model because of the difference in the required sample size for evaluation. Candidate and selected covariates are provided in Supplementary Table 3.

### Sensitivity analysis

We conducted four sensitivity analyses. First, we created a placebo treatment by post hoc assignment of 2000 random binary variables to ensure the lack of heterogeneity in the absence of treatment effects. Second, we evaluated the model performance in 2000 random non-temporal splitting of datasets. Third, we created samples excluding emergency surgery (n = 217; 10.7%) to ensure that the heterogeneity was not due to inadequate presurgical evaluation. Fourth, we evaluated the AUUC excluding sample proportion below 0.3 or 0.4 in the uplift curve to ensure that the result was not due to insufficient confounder adjustment in a small subsample size.

### Statistical analysis

All analyses were performed using Python version 3.8.9 and the following add-on libraries: Scikit-learn package version 0.24.2 for all machine learning models, EconML^40^ version 0.12.0 for all CATE models, and SHAP^27^ version 0.39.0 for model interpretation. A two-sided *P* value of < 0.05 was considered significant.

## Supplementary Information

Supplementary Information.

Supplementary Table 3.

## Data Availability

The deidentified data are available from the corresponding author upon reasonable request.
