# Supplementary material for: Machine learning-based causal models for predicting the response of individual patients to dexamethasone treatment as prophylactic antiemetic
Source: Sci Rep. 2023 May 9;13:7549. doi: 10.1038/s41598-023-34505-0 (PMC10169123; doi:10.1038/s41598-023-34505-0)
Supplement: Supplementary file 1 — Supplementary Information. [file 41598_2023_34505_MOESM1_ESM.docx]

**SUPPLEMENTARY INFORMATION FOR**

Machine Learning-Based Causal Models for Predicting the Response of Individual Patients to Dexamethasone Treatment as Prophylactic Antiemetic

**Taisuke Mizuguchi^1^*, Shigehito Sawamura^1^**

*^1^Department of Anesthesia, Teikyo University, Tokyo, Japan*

**Corresponding author: Taisuke Mizuguchi (mizuguchi@med.teikyo-u.ac.jp)*

**Supplementary Figure 1. Distribution of the propensity score**

**Supplementary Figure 2. Uplift curve evaluation**

(**a**) (**c**) (**e**). The uplift curve of conditional average treatment effect (CATE) models and risk prediction models. The x-axis is the sample proportion *k*/*n* (*k* = 1, 2, . . . , *n*; *n*, total sample size), where each subsample consists of samples with top *k* values of estimated CATE. The y-axis is the estimated difference in postoperative nausea and vomiting (PONV) events between the treated and untreated groups, calculated from the observed outcome in each subsample. Negative values correspond to the estimated number of PONV events reduced by the treatment. The baseline serves as a reference, plotting the expected uplift curve value when the subsamples consist of random CATE. If the CATE value is estimated correctly, a greater reduction in the PONV event should be observed in the uplift curve compared to the baseline. (**b**) (**d**) (**f**). The area under the uplift curve (AUUC) is calculated from the uplift curve. A greater positive AUUC indicates better model performance in identifying the patients likely to respond to the treatment. The AUUC of a null model is zero. The error bars indicate 95% confidence intervals obtained from 2,000 cycles of bootstrap resampling. Abbreviations: IPW, inverse probability weighting; DML, double machine learning; DR, doubly robust; GRF, generalized random forest.

**Supplementary Figure 3. Sensitivity analysis (double machine learning)**

The area under the uplift curve (AUUC) is calculated from the uplift curve. For placebo, conditional average treatment effect (CATE) is estimated using 2,000 patterns of random binary variables assigned *post hoc* as treatment instead of dexamethasone. Random split evaluated the model using 2,000 patterns of non-temporal splitting of the dataset. Elective surgery uses the samples excluding emergency surgeries. Uplift curve sample proportion ≥0.3 or 0.4 evaluate the AUUC excluding sample proportion below 0.3 or 0.4 in the uplift curve, respectively.

**Supplementary Figure 4. Sensitivity analysis (doubly robust learner)**

**Supplementary Figure 5. Sensitivity analysis (forest double machine learning)**

**Supplementary Figure 6. The study flowchart**

**Supplementary Table 1. Category distribution of PONV events ^a^**

|  | Overall (n=2,026) | Training/Validation (n=1,219) | Test (n=807) |
| --- | --- | --- | --- |
| Male | 183 (17.5) | 117 (18.3) | 66 (16.1) |
| Female | 294 (30.0) | 173 (29.8) | 121 (30.4) |
| Smoker | 70 (17.2) | 42 (16.7) | 28 (18.1) |
| Ex-smoker | 120 (22.7) | 68 (23.2) | 52 (22.0) |
| Non smoker | 287 (26.3) | 180 (26.7) | 107 (25.7) |
| PONV or motion sickness history | 81 (41.8) | 49 (43.0) | 32 (40.0) |
| Hypertension | 143 (21.6) | 86 (22.9) | 57 (19.9) |
| Diabetes | 71 (23.2) | 44 (23.9) | 27 (22.1) |
| Psychiatric disease | 20 (19.6) | 12 (17.4) | 8 (24.2) |
| Malignancy | 154 (25.9) | 87 (27.3) | 67 (24.4) |
| History of stroke | 29 (17.9) | 21 (19.6) | 8 (14.5) |
| Athema | 35 (38.5) | 20 (36.4) | 15 (41.7) |
| COPD | 46 (23.7) | 30 (20.1) | 16 (35.6) |
| Coronary disease | 7 (14.3) | 3 (11.5) | 4 (17.4) |
| Post PCI or CABG | 18 (21.7) | 14 (27.5) | 4 (12.5) |
| Asynergy | 16 (25.4) | 10 (27.8) | 6 (22.2) |
| ASA-PS 1 | 146 (26.2) | 94 (25.3) | 52 (28.0) |
| ASA-PS 2 | 288 (23.4) | 167 (24.2) | 121 (22.2) |
| ASA-PS 3 | 43 (18.2) | 29 (18.2) | 14 (18.2) |
| TIVA | 53 (19.9) | 29 (20.3) | 24 (19.5) |
| Peripheral nerve block | 72 (22.0) | 39 (22.0) | 33 (21.9) |
| Epidural anesthesia | 73 (27.5) | 44 (30.3) | 29 (24.2) |
| Continuous opioid infusion | 192 (27.3) | 125 (30.1) | 67 (23.2) |
| Droperidol bolus | 58 (24.4) | 27 (20.6) | 31 (29.0) |
| Dexamethasone bolus | 166 (22.0) | 96 (21.9) | 70 (22.0) |
| Elective_surgery | 438 (24.2) | 270 (24.6) | 168 (23.7) |
| Emergency surgery | 39 (18.0) | 20 (16.7) | 19 (19.6) |
| ICU admission | 128 (28.4) | 87 (28.1) | 41 (29.1) |

Abbreviations: PONV, postoperative nausea and vomiting; COPD, chronic obstructive pulmonary disease; PCI, percutaneous Coronary Intervention; CABG, coronary artery bypass graft; ASA-PS, American Society of Anesthesiologists Physical Status; TIVA, total intravenous anesthesia; ICU, intensive care unit.

a Data are expressed as No. of PONV events (%).

**Supplementary Table 2. Performance evaluation of risk prediction models**

|  | AUROC | Sensitivity | Specificity | Accuracy |
| --- | --- | --- | --- | --- |
| Optimized risk model | 0.714 | 0.706 | 0.874 | 0.637 |
| Base risk model | 0.635 | 0.631 | 0.843 | 0.605 |
